# Association between anaemia and osteoporosis: a systematic review and meta-analysis

**DOI:** 10.1080/07853890.2025.2610878

**Published:** 2026-01-06

**Authors:** Alveron Andreas Tear, Florencia Anastasia Tesno, Haneira Shofiadeita, Gading Marcell Bunga, Hamidah Nur Taqiya, Najwa Audrey Sanditha, Ayesha Humayra Fayyaza, Samantha Kerenhapukh Tiurlina Tambunan, Gisela Kayla Wangsa, Qanita Reezqi Fatimah, Istiqomah Istiqomah, Widya Wibawanty, Leonardo Lubis

**Affiliations:** a Faculty of Medicine, Universitas Padjadjaran, Bandung, Indonesia; b Department of Biomedical Sciences, Faculty of Medicine, Universitas Padjadjaran, Bandung, Indonesia

**Keywords:** Anaemia, haemoglobin, osteoporosis, risk factor

## Abstract

**Background:**

Osteoporosis significantly impacts global morbidity. Recent evidence suggests anaemia may contribute to osteoporosis risk. This systematic review and meta-analysis investigates this association.

**Methods:**

PubMed, Scopus, EBSCO, and ScienceDirect were searched for papers. Studies with definition of anaemia and assessing osteoporosis outcomes were included. Meta-analysis utilized random-effects models (DerSimonian-Laird method), and study quality was assessed *via* Newcastle-Ottawa Scale (NOS). Analyses were performed using R Studio.

**Result:**

Eighteen studies (861,540 participants) were analyzed. Anaemia significantly increased osteoporosis risk in univariate analysis (OR 1.62; 95% CI 1.33–1.98; *p* < 0.001), despite high heterogeneity (I^2^ = 92.7%). The results remain significant in studies that reported multivariate analysis (OR 2.01; 95% CI 1.26–3.21; *p* = 0.004). Sensitivity analyses confirmed the robustness of our result.

**Conclusion:**

Anaemia significantly associated with osteoporosis, emphasizing the need for targeted screening in anaemic individuals. Further studies should consider incorporating anaemia into osteoporosis and fracture prediction tools.

## Introduction

Osteoporosis is a systemic skeletal disorder characterized by reduced bone mineral density (BMD) and altered bone microarchitecture, increasing bone fragility and fracture risk [1–3]. Globally, over 200 million individuals are affected, with approximately 9 million osteoporotic fractures occurring annually [4]. Osteoporotic fractures significantly reduce quality of life and increase morbidity and mortality, especially among older adults [1].

Haemoglobin (Hb), the primary protein transporting oxygen to tissues, is suggested to influence bone health. Anaemia is one of the most common blood disorders worldwide, contributing to significant morbidity and mortality. Globally, it affects nearly a quarter of the population, approximately 1.9 billion people [5]. It is highly prevalent in older adults, which are populations with higher osteoporosis risk and increased fracture susceptibility [6]. Chronic anaemia can induce hypoxaemia, increasing osteoclastic activity and disrupting bone remodelling, thereby accelerating bone mass loss [7].

Recent studies support this association; increased Hb levels correlate with reduced osteoporosis risk, whereas anaemia significantly increases osteoporosis incidence [7]. Low Hb levels are linked to reduced BMD and nearly double the risk of osteoporotic fractures. Thus, haemoglobin status may be a valuable marker for assessing osteoporosis and fracture risk in vulnerable groups. Therefore, this study aims to analyze the association between anaemia and the development of osteoporosis in adults.

## Methods

### Search strategy

The systematic review protocol was registered in PROSPERO (registration number: CRD420251049038) and conducted following PRISMA guidelines. PubMed, Scopus, ScienceDirect, and EBSCO databases were comprehensively searched without language or publication year restrictions, up to 9^th^ May 2025, using the terms: Anaemia, Osteoporosis, and Risk Factor (Supplementary Table S1).

### Study selection

Articles were included if they were observational studies (cohort or case-control), original research, conducted in adults or older adults, included any type of anaemia, and reported outcomes as odds ratio or hazard ratio. Exclusion criteria were case reports or case series, *in vitro*/*in vivo* studies, studies analyzing anaemia without proper definition of anaemia, and outcomes not specific to osteoporosis. Study selection was conducted by ten authors that were separated into five teams to ensure each article was selected by at least 2 independent authors (AAT, FAT, HS, GMB, HNT, NAS, AHF, SKT, GKW, QRF). Conflict was resolved with the help of a third author (II, WW).

### Data extraction and quality assessment

Ten authors that were separated into five teams (AAT, FAT, HS, GMB, HNT, NAS, AHF, SKT, GKW, QRF) extracted the characteristic of included studies (author, year, country, sample size, study design, age, haemoglobin cut-off for anaemia, type of anaemia, follow up duration) and assessed study quality. Newcastle-Ottawa Scale (NOS) was used to assess the quality of each study, which awards up to nine stars across selection, comparability, and outcome/exposure domains. Studies were classified as good quality (3–4 stars in the selection domain, 1–2 stars in the comparability domain, and 2–3 stars in the outcome/exposure domain), fair quality (2 stars in the selection domain, 1–2 stars in the comparability domain, and 2–3 stars in the outcome/exposure domain), and poor quality (0–1 star in the selection domain, 0 stars in the comparability domain, or 0–1 stars in the outcome/exposure domain) according to the Agency for Healthcare Research and Quality (AHRQ). Disagreements were resolved by a third author (LL) [8].

### Data synthesis

Odds ratios, and their 95% confidence intervals were extracted; if unavailable, odds ratios were calculated using provided data. Study heterogeneity was assessed using the I^2^ statistic (>50%) and p-value (<0.05). Subgroup analyses were performed based on anaemia type, age (geriatric-exclusive defined as adults aged >65 years versus non-geriatric), and comorbidities. All statistical analyses were conducted using R Studio.

## Results

### Study selection

The study selection process began with the identification of 3,855 records through electronic database searches, which included PubMed (*n* = 640), Scopus (*n* = 2,656), EBSCO (*n* = 434), and ScienceDirect (*n* = 125). Following the removal of 1,089 duplicate records, 2,767 records were screened based on titles and abstracts. During this screening phase, 2,673 records were excluded due to irrelevance to the study objective or design.

**Figure 1.**
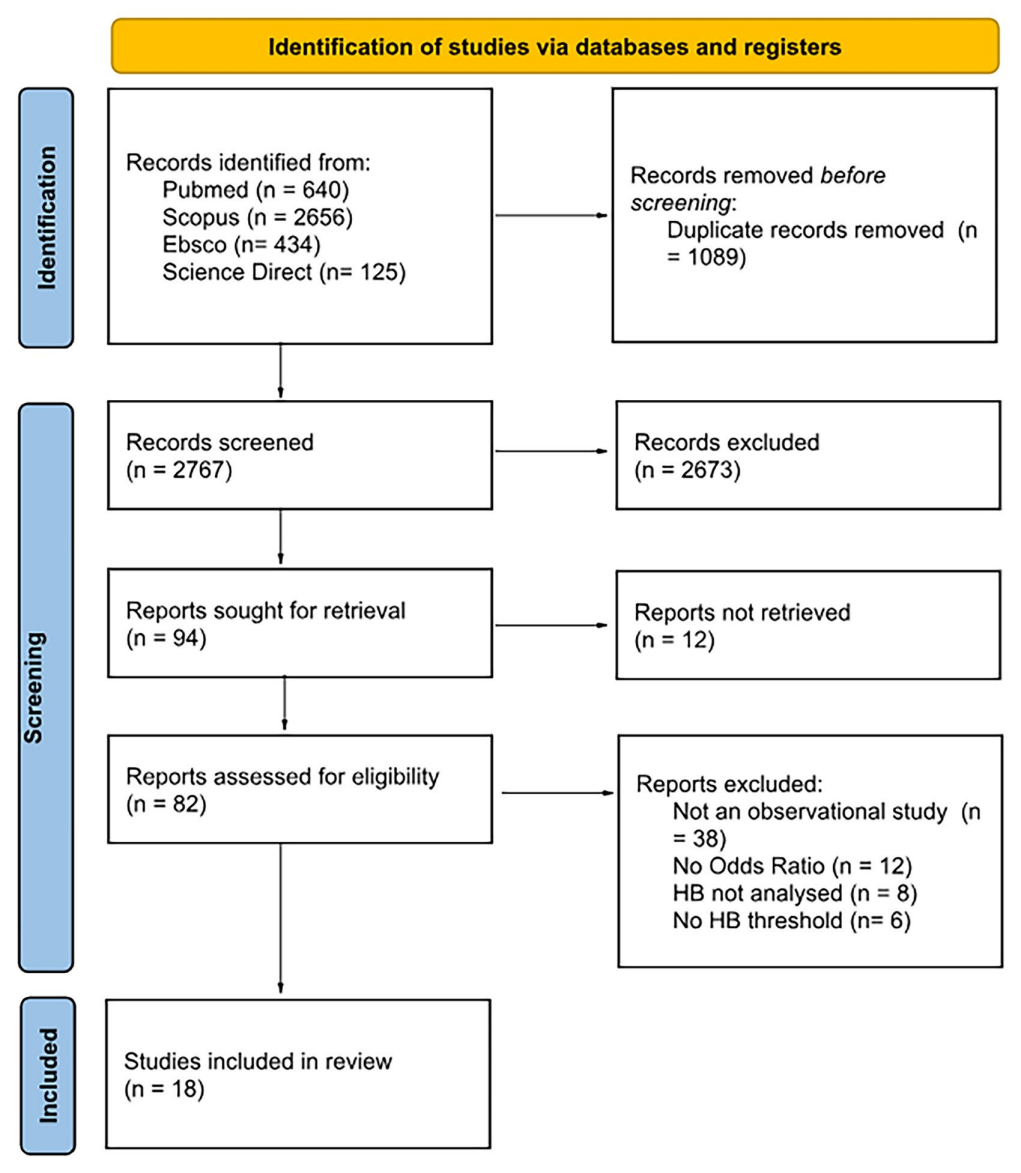
PRISMA flowchart of study selection process.

The remaining 94 studies were sought for full-text retrieval; however, 12 of these could not be retrieved. A total of 82 full-text articles were assessed for eligibility. Of these, 64 were excluded for the following reasons: 38 were not observational studies, 12 did not report odds ratios, 8 did not analyse anaemia, and 6 did not define anaemia. Ultimately, 18 studies fulfilled the inclusion criteria and were included in the final meta-analysis (Figure 1).

### Study characteristic

From the 18 studies included, the publication time ranged from 2007 to the latest in 2024. There were 861,540 total patients who were the subject of these studies. There were 8 studies that were cohort, 7 studies that were cross-sectional, and 1 study that was a case control. Geographically, the studies were conducted in China, Mexico, Korea, Taiwan, Iran, Netherland, Sweden, England, Wales, and Scotland.

These studies were each conducted in the adult and geriatric population. Cut-offs of haemoglobin mentioned in the studies ranged from 11 to 14 g/dL. The types of the anaemia mentioned in the studies were mostly unknown, but there were 2 of the studies that mentioned haemolytic anaemia and iron deficiency anaemia as the type of anaemia. Follow up duration of most of the studies were also unknown. Nevertheless, the known follow up duration of the 6 out of 16 studies ranged from 2.4–14 years. The median NOS score was 6 (Table 1).

**Table 1.** Study characteristics.

Author, year	Country	Sample size	Study design	Age	Cut-off	Type of anaemia	Follow up duration	Definition of osteoporosis	NOS	Quality
Ran Cui. 2021 [9]	China	2336	Cohort	Adult	Men: ≤ 12 g/dL Women: ≤ 11 g/dL	N/A	N/A	Low BMD ( ≤ − 2.5 SD)	6	Good
Heidari Behzad. 2016 [10]	Iran	553	Cross-sectional	Geriatric	<13 g/dL	N/A	4.6 years	Low BMD (≤ − 2.5)	9	Good
Huang Yanjun, 2024 [11]	Wales, England, Scotland	452 778	Cohort	Adult & geriatric	Men: <13 g/dL Women: <12 g/dL	N/A	5.84 years	Low BMD ( ≤ − 2.5 SD) or osteoporotic fractures	9	Good
Jaiswal Raju, 2023 [7]	Sweden	2778	Cohort	Geriatric	<12 g/dL	N/A	6.4–10 years	Low BMD ( ≤ − 2.5 SD) or osteoporotic fractures	9	Good
Batún-Garrido JAJ. 2018 [12]	Mexico	122	Cohort	Adult	< 12 g/dL	N/A	N/A	Low BMD (≤ − 2.5)	6	Good
Kim SY. 2021 [6]	Korea	139520	Case control	Adult	Men: <13 g/dL Women: <12 g/dL	N/A	N/A	Low BMD ( ≤ − 2.5 SD) or osteoporotic fractures	6	Fair
Jung Sub, Lim. 2007 [13]	South Korea	133	Cross-sectional	Adult	Men: < 13 g/dL Women: < 11 g/dL	N/A	2.7 ± 2.4 years	Low BMD ( ≤ − 2.5 SD) or osteoporotic fractures	7	Good
Liu Y. 2023 [14]	China	1048	Cross-Sectional	Geriatric	Men: ≤14.3 g/dL Women: 13 g/dL	N/A	N/A	Low BMD (≤ − 2.5)	4	Poor
Yuh Hwan, Oh. 2017 [15]	Korea	1574	Cross-sectional	Adult	Men: <13 g/dL Women: <12 g/dL	N/A	N/A	Low BMD	6	Fair
Mei-Lien Pan. 2017 [16]	Taiwan	214506	Cohort	Adult	Men: <13 g/dL Women: <12 g/dL	Iron deficiency anemia	14 years	Low BMD ( ≤ − 2.5 SD) or osteoporotic fractures	8	Good
Rutten Erica P. A., 2012 [17]	Netherland	321	Cohort	Adult	Men: < 13 g/dL Women: < 12 g/dL	N/A	N/A	Low BMD (≤ − 2.5)	6	Fair
Shi, Leiyu. 2021 [18]	Taiwan	11210	Cohort	Adult	N/A	Hemolytic anemia	14 years	Low BMD ( ≤ − 2.5 SD) or osteoporotic fractures	6	Good
Wang M. 2022 [19]	China	302	Cohort	Adult	Men: 13 g/dL Women: 12 g/dL	N/A	N/A	Low BMD (≤ − 2.5)	7	Good
Xiu S. 2019 [20]	China	370	Cross-sectional	Geriatric	Men: <13 g/dL Women: <12 g/dL	N/A	N/A	Low BMD ( ≤ − 2.5 SD) or osteoporotic fractures	5	Fair
Xiu Shuangling. 2022 [21]	China	573	Cross sectional	Geriatric	Men: <13 g/dL Women: <12 g/dL	N/A	N/A	Low BMD ( ≤ − 2.5 SD) or osteoporotic fractures	8	Good
Ye, Tingting. 2022 [22]	China	495	Cross-sectional	Adult	Men: <13 g/dL Women: <12 g/dL	N/A	N/A	Low BMD ( ≤ − 2.5 SD) or osteoporotic fractures	7	Fair
Shin DW. 2019 [23]	South Korea	31916	Cohort	Adult	< 12 g/dL	N/A	10 years	Osteoporotic fracture	8	Good
Kristjansdottir HL. 2022 [24]	Sweden	1005	Cohort	Geriatric	<13 g/dL	N/A	16 years	Osteoporotic fracture	9	Good

### Findings

**Figure 2.**
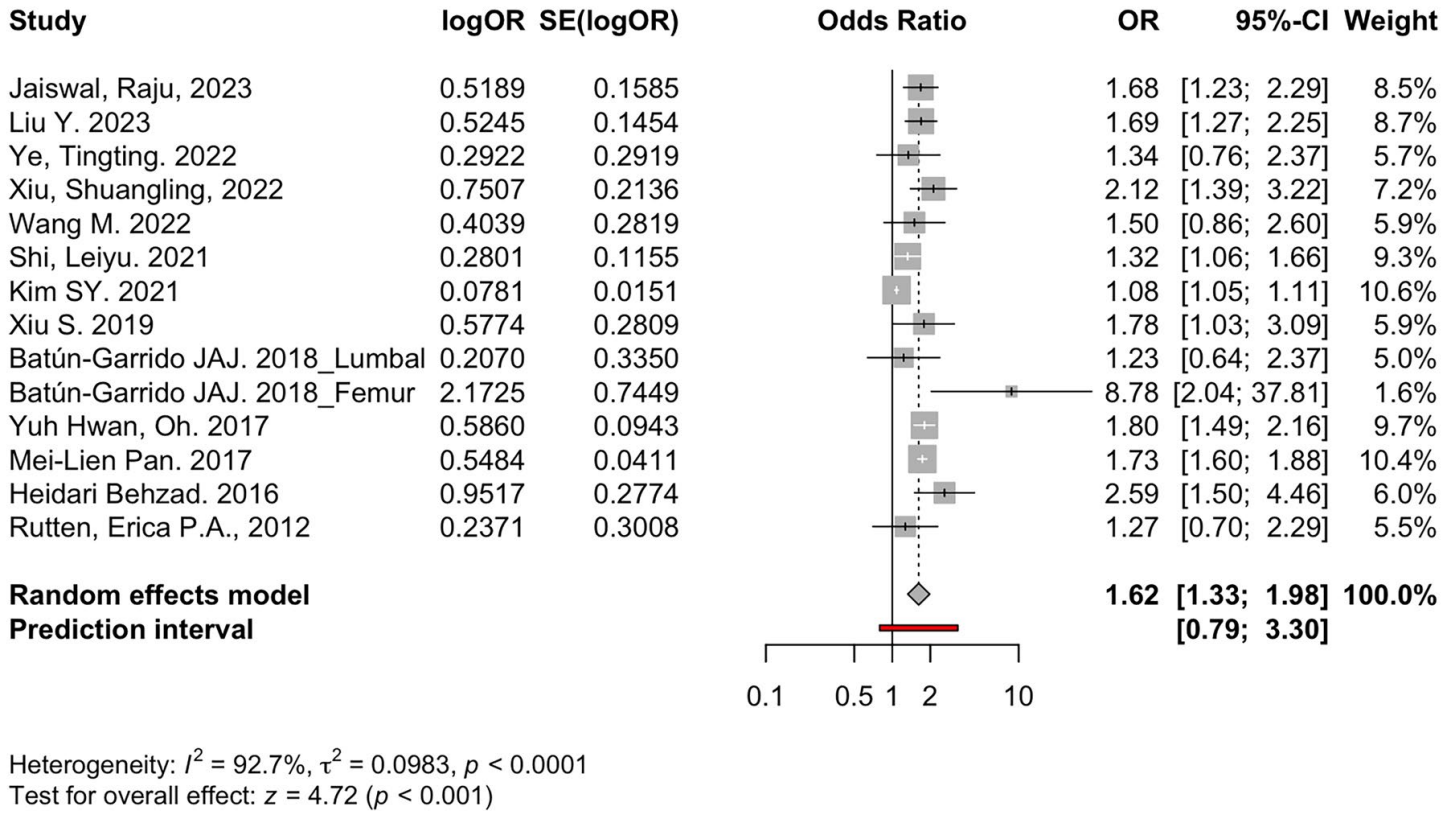
Association between anaemia and the risk of osteoporosis among studies that reported univariate odds ratios (OR).

The forest plot (Figure 2) demonstrates a pooled OR of 1.62 with a 95% confidence interval (CI) of 1.33 to 1.98 (*p* < 0.001), indicating that individuals with anaemia have a 62% higher odds of osteoporosis compared to those without anaemia. However, the prediction interval ranges from 0.79 to 3.30, indicating that while the average effect is positive, future studies may find a weaker or even non-significant relationship. The analysis shows high heterogeneity among the included studies (I^2^ = 92.7%, *p* < 0.0001), reflecting substantial variability in study outcomes that could be due to differences in populations, methodologies, or other contextual factors.

While some studies reported strong associations such as Heidari Behzad (2016) with an OR of 2.59 and Batún-Garrido (2018, Femur) with an OR of 8.78 others showed weaker or borderline significant effects. Notably, studies like Kim SY (2021), which contributed the most weight due to high precision, reported a modest effect (OR 1.08). Overall, the findings support the hypothesis that anaemia is associated with increased odds of the adverse outcome, but the wide variability among studies underscores the need for further investigation into potential moderators or confounding factors.

**Figure 3.**
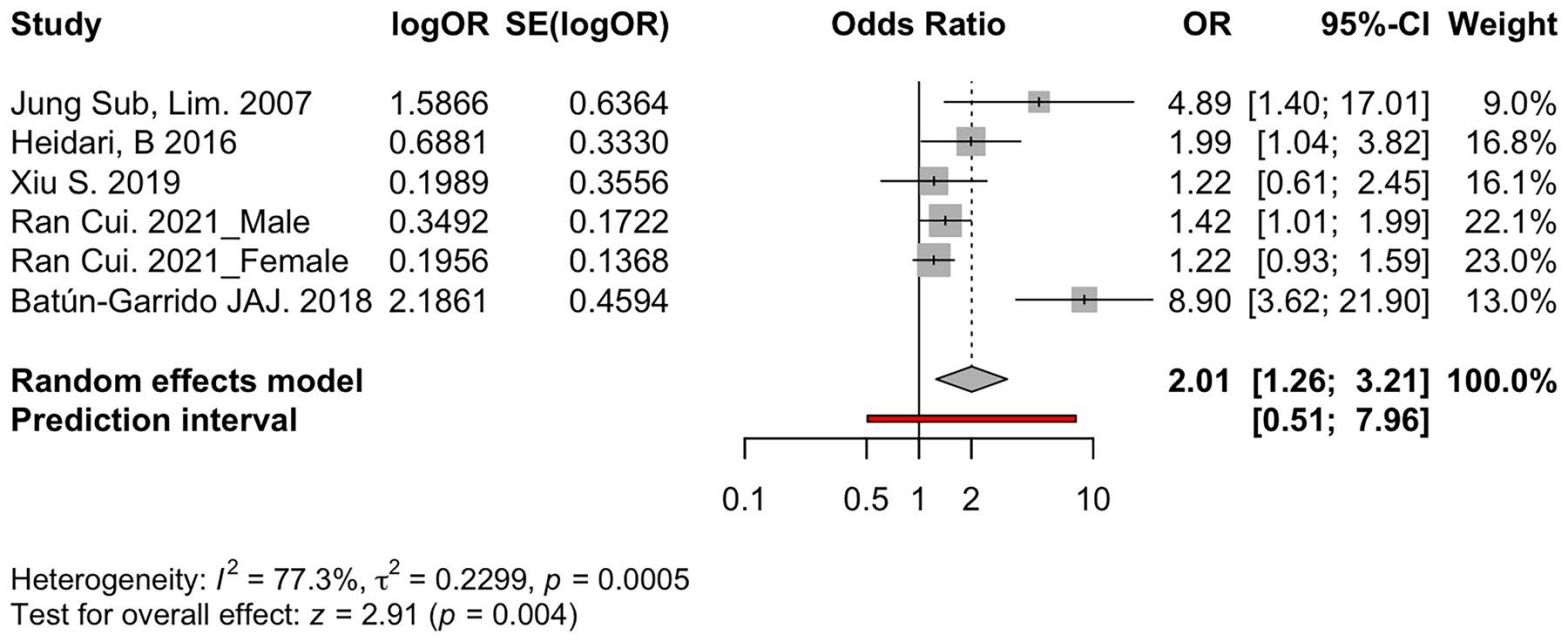
Association between anaemia and the risk of osteoporosis among studies that reported multivariate odds ratios (OR).

The forest plot (Figure 3) demonstrates a pooled OR of 2.01 with a 95% confidence interval (CI) of 1.26 to 3.21 (*p = 0.004*). The analysis shows high heterogeneity among the included studies (I^2^ = 77.3%, *p = 0.0005*), reflecting substantial variability in study outcomes that could be due to differences in populations, methodologies, or other contextual factors.

**Figure 4.**
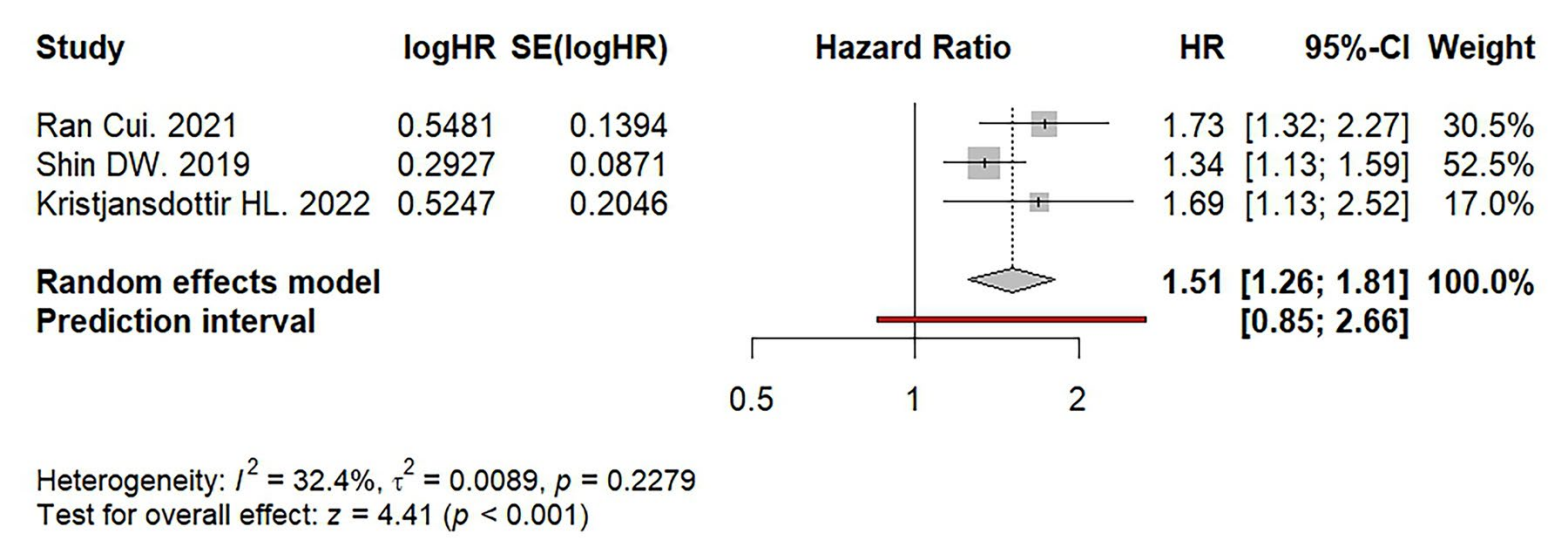
Association between anaemia and the risk of osteoporotic fractures among studies that reported multivariate hazard ratio (HR).

The forest plot (Figure 4) demonstrates a significant association between anaemia and osteoporotic fracture with pooled HR of 1.51 with a 95% confidence interval (CI) of 1.26 to 1.81 (*p* < 0.001). The heterogeneity between studies was low (I^2^= 32.4%, *p* = 0.2279).

### Sensitivity analysis

This forest plot (Supplementary Figure S1) presents a sensitivity analysis assessing the robustness of the overall effect size by sequentially omitting each study from the meta-analysis among studies that reported univariate odds ratio. The overall pooled odds ratio (OR) remains stable at 1.62 [95% CI: 1.33–1.98], with a statistically significant p-value (< 0.0001), indicating a consistent and robustness association. When individual studies are excluded, the OR values range narrowly from 1.57 to 1.68, and all 95% confidence intervals remain statistically significant, further confirming the reliability of the results.

Notably, a single study (Kim SY, 2021) appears to have a particularly strong influence on the pooled estimate. When omitted, the odds ratio increases to 1.68 [95% CI: 1.52–1.86], the highest among all iterations, with a marked reduction in heterogeneity to I^2^ = 26.1%. This suggests that the inclusion of this study may be the main source of heterogeneity.

This forest plot (Supplementary Figure S2) presents a sensitivity analysis evaluating the robustness of the pooled effect by sequentially omitting each study. The overall OR remains stable at 2.01 [95% CI: 1.26–3.21, *p* = 0.004], indicating a consistent and significant association. Excluding individual studies yields ORs ranging from 1.45 to 2.44, all statistically significant, confirming result reliability. Notably, removing Batún-Garrido JAJ (2018) lowers the OR to 1.45 and reduces heterogeneity (I^2^ = 35.0%), suggesting its substantial contribution to interstudy heterogeneity. Conversely, omitting Ran Cui. 2021_Female raises the OR to 2.44, with heterogeneity largely unchanged. Overall, no single study unduly influenced the findings.

### Subgroup analysis

The forest plot below (Supplementary Figure S3) presents a subgroup analysis comparing studies that are exclusively geriatric (‘geriatric_exclusive = Yes’) with those that are not (‘geriatric_exclusive = No’) in relation to an outcome measured by univariate odds ratio (OR). The pooled OR for the geriatric-exclusive subgroup is 1.83 [95% CI: 1.55–2.17], indicating a statistically significant positive effect (*p* < 0.001) with no observed heterogeneity (I^2^ = 0%). In contrast, the non-geriatric-exclusive subgroup has a pooled OR of 1.47 [95% CI: 1.15–1.89], also statistically significant (*p* = 0,002), but with substantial heterogeneity (I^2^ = 94.6%). The test for subgroup differences is not statistically significant ((χ^2^ = 2.03, *p* = 0.1544), indicating no strong evidence of effect modification by subgroup classification. Overall, the results suggest a stronger and more consistent effect in studies focused exclusively on geriatric populations, although the difference between subgroups is not statistically significant.

The forest plot below (Supplementary Figure S4) presents a subgroup analysis comparing studies that are exclusively geriatric (‘geriatric_exclusive = Yes’) with those that are not (‘geriatric_exclusive = No’) in relation to an outcome measured by multivariate odds ratio (OR). The pooled OR for the geriatric-exclusive subgroup is 1.58 [95% CI: 0.98–2.55], indicating a positive but borderline statistically significant effect (*p = 0.060*) with minimal heterogeneity (*I^2^ = 0.9%*). In contrast, the non-geriatric-exclusive subgroup has a pooled OR of 2.41 [95% CI: 1.23–4.71], which is statistically significant (*p = 0.010*) and shows substantial heterogeneity (*I^2^ = 85.6%*). The test for subgroup differences is not statistically significant (χ^2^ = 1.00, *p = 0.3177*), indicating no strong evidence of effect modification by subgroup classification. Overall, the results suggest a stronger and statistically robust effect in the non-geriatric-exclusive studies, though the observed difference between the subgroups is not significant.

This forest plot (Supplementary Figure S5) presents a subgroup analysis among studies that reported univariate odds ratio based on anaemia type: unspecified type (N/A), haemolytic anaemia, and iron deficiency anaemia. The largest subgroup, Type = N/A includes multiple studies and shows a pooled odds ratio (OR) of 1.65 [95% CI: 1.30–2.09], indicating a statistically significant effect (p, 0,001) with moderate heterogeneity (I^2^ = 85.3%). The haemolytic anaemia subgroup includes a single study (Shi, 2021) with an OR of 1.32 [95% CI: 1.06–1.66], showing a significant effect. The iron deficiency anaemia subgroup (Mei-Lien Pan, 2017) also shows a significant OR of 1.73 [95% CI: 1.60–1.88]. The test for subgroup differences (χ^2^ = 4.81, *p* = 0.0903) is not statistically significant, indicating no strong evidence that the effect differs by anaemia type. Therefore, regardless of anaemia type, the exposure appears to be associated with increased odds of the outcome, with the strongest and most consistent effect observed in studies where the anaemia type was not specified. In studies that reported multivariate odds ratio, all of them did not specify the type of anaemia, therefore subgroup analysis could not be performed.

This subgroup meta-analysis (Supplementary Figure S6) explores the association between anaemia and the risk of osteoporosis based on different comorbid conditions using univariate odds ratios (ORs). The pooled OR across all studies is 1.62 (95% CI: 1.33 to 1.98, *p* < 0.001), indicating that anaemia is significantly associated with an increased risk of osteoporosis. Subgroup analyses were performed based on the exclusive presence of comorbidities: no comorbidity, type 2 diabetes mellitus (T2DM), rheumatoid arthritis (RA), and chronic obstructive pulmonary disease (COPD).

In studies without specific comorbidities (NA subgroup), the pooled OR is 1.59 (95% CI: 1.25 to 2.02), with substantial heterogeneity (I^2^ = 95.6%, *p* < 0.001). In the T2DM subgroup, the OR is higher at 1.80 (95% CI: 1.35 to 2.40), with no observed heterogeneity (I^2^ = 0%), suggesting a consistent association across studies. For the RA subgroup, the pooled OR is 2.94 (95% CI: 0.43 to 19.90), but this estimate has wide confidence intervals and moderate heterogeneity (I^2^ = 82.7%), indicating less precision and consistency. The COPD subgroup shows a non-significant association with an OR of 1.27(95% CI: 0.70 to 2.29). Despite differences in effect sizes across comorbidity groups, the test for subgroup differences is not statistically significant (*p* = 0.6686), suggesting that the association between anaemia and osteoporosis risk does not significantly differ by comorbidity status.

This subgroup meta-analysis (Supplementary Figure S7) explores the association between anaemia and osteoporosis based on different exclusive comorbid conditions in multivariate analyses. The pooled OR across all studies is 2.01 (95% CI: 1.26 to 3.21, *p* = 0.004), indicating a statistically significant association. Subgroup analyses were performed based on the exclusive presence of comorbidities: cancer, rheumatoid arthritis (RA), type 2 diabetes mellitus (T2DM), chronic obstructive pulmonary disease (COPD), and no comorbidity (NA). The test for subgroup differences is statistically significant (χ^2^ = 21.49, *p* = 0.0003), suggesting that the association between the exposure and outcome differs significantly across comorbidity subgroups. These findings highlight that the presence and type of comorbid conditions may modify the strength of the association, with the most pronounced effects observed in studies involving cancer or RA.

We also performed subgroup meta-analysis comparing studies that used WHO definition of anaemia with those that used other definitions of anaemia in multivariate and univariate analyses (Supplementary Figures S9 and S10). In studies that used univariate analysis, both studies that used WHO definition of anaemia (*p* < 0.001*)* and other definitions of anaemia (*p* < 0.001) showed significant association with osteoporosis. However, in studies that used multivariate analysis, studies that used WHO guidelines showed non-significant association (*p* = 0.238).

### Assessment of publication bias

The funnel plot (Supplementary Figure S8) illustrates the assessment of publication bias in the meta-analysis examining the association between anaemia and the risk of osteoporosis using univariate odds ratios (ORs). Each dot represents an individual study, with the x-axis showing the OR and the y-axis displaying the standard error. Ideally, in the absence of publication bias, the studies would be symmetrically distributed around the pooled effect size, forming a funnel-shaped pattern.

However, in this plot, there appears to be asymmetry, with fewer studies appearing on the left side, indicating a potential absence of studies with small or non-significant effects. Egger’s test for funnel plot asymmetry shows a statistically significant result (*p* = 0.0179), suggesting the presence of small-study effects or publication bias. In contrast, Begg’s test yields a non-significant result (*p* = 0.1124), providing weaker evidence of publication bias. Overall, the findings suggest that publication bias may be present, as supported by the significant Egger’s test and the visual asymmetry of the plot.

## Discussion

This meta-analysis demonstrates a significant association between anaemia and an increased risk of osteoporosis. Individuals with anaemia were found to have a higher risk of osteoporosis compared to those with normal haemoglobin levels [25]. Sensitivity analysis confirmed the robustness of the findings. These results suggest that anaemia has systemic effects that negatively impact bone health [25].

This study expands upon previous research by including a broader population and highlights that anaemia, particularly iron deficiency anaemia, may contribute to bone loss through mechanisms such as tissue hypoxia, oxidative stress, and disrupted bone metabolism [25,26]. Through a number of interrelated mechanisms, including iron deficiency, iron overload, oxidative stress, FGF23-mediated mineral disturbances, and compromised muscle function, iron dysregulation leads to bone fragility. Osteoporosis and fracture risk are increased by both low and high iron levels, according to experimental and clinical research [27–29]. This is especially true for people who also have vitamin D deficiency or malnutrition. These mechanisms contribute to the explanation of why, in a variety of populations, anaemia is consistently linked to lower bone mineral density and a higher incidence of fragility fractures [30,31]. Therefore, early detection and appropriate management of anaemia may serve as an important strategy in preventing osteoporosis and related fractures.

Anaemia, regardless of its underlying cause, often leads to tissue hypoxia due to reduced haemoglobin levels [26,31]. Hypoxia has been shown to stimulate bone resorption through multiple mechanisms, induction of osteoclastogenesis, hypoxia increases the expression of hypoxia-inducible factors (HIFs), which in turn enhance the differentiation and activity of osteoclasts, the cells responsible for bone breakdown. Anaemia-induced hypoxia promotes the formation of an acidic microenvironment, which favours osteoclastic activity and the dissolution of bone matrix and impairs the proliferation and differentiation of osteoblasts, the cells responsible for bone formation [32,33]. This results in a net loss of bone mass and reduced bone mineralization [25]. In addition to these hypoxia-mediated changes, anaemia, particularly when accompanied by iron deficiency, can enhance the uptake of toxic metals that further compromise bone integrity. Iron deficiency increases the density of transferrin receptors (TfR1), allowing greater intracellular entry of aluminium, which is osteotoxic even at low doses and contributes to impaired osteoblast function and ferroptotic cell injury [34]. At the same time, hypoxia has been shown to up-regulate divalent metal transporter-1 (DMT-1), facilitating increased cadmium uptake, a metal strongly associated with trabecular deterioration, fragility fractures, and osteoporosis [35,36]. These combined effects intensify bone remodelling imbalance in anaemic individuals and amplify pathways that accelerate skeletal weakening. Another critical link between anaemia and osteoporosis lies in bone marrow physiology. Blood loss and anaemia stimulate haematopoiesis, the process of generating new blood cells by increasing levels of haematopoietic growth factors. These growth factors not only boost the proliferation of haematopoietic cells but also inadvertently influence bone cell activity, osteoclast proliferation, haematopoietic stem cells give rise to osteoclasts, and increased haematopoietic activity leads to enhanced bone resorption [37]. Osteoblast progenitor activation, imbalance between the rates of osteoclast and osteoblast proliferation favours bone loss and, expansion of haematopoietic territories within the bone marrow, often at the expense of bone trabeculae, resulting in weaker bone structure and contributing to osteoporosis [15].

This meta-analysis presents few limitations that should be carefully considered. First, the substantial heterogeneity observed among the included studies (I^2^ = 92.7%, *p* < 0.0001), shows that it might reflect variations in study design, population characteristics and the definitions of anaemia and osteoporosis. Although subgroup analyses were conducted to explore potential sources of variability, not all contributing factors could be fully identified. Second, there is a possibility of publication bias. Egger’s test indicated a statistically significant result (*p* = 0.0179), and the visual assessment of the funnel plot also shows asymmetry. This indicated that smaller studies with non-significant findings might be still underrepresented.

Lastly, there is variability in how anaemia is defined across the studies, including differences in haemoglobin cut-off values and lack of specification regarding anaemia subtypes in several cases. This inconsistency might limit the precision of our subgroup analyses and affect the generalizability of our findings. Several included studies did not employ the WHO-defined criteria for anaemia, therefore a severity stratification approach correlating with osteoporosis and fractures risk could be adopted. Despite these limitations, the results of this meta-analysis have offered valuable insights and highlights the potential relationship between haemoglobin levels and osteoporosis. We encourage further studies with standardized methodologies and broader population representation to strengthen these findings.

## Conclusion

Anaemia is significantly associated with osteoporosis, therefore screening for osteoporosis should be prioritized for adults with anaemia. Further studies should be conducted to integrate anaemia in osteoporosis and fracture prediction tools to provide more comprehensive data.

## Supplementary Material

Supplemental Material

Supplementary Figure 10.jpeg

Supplementary Figure 3.jpeg

Supplementary Figure 2.jpeg

Supplementary_PRISMA_2020_checklist.docx

Supplementary Figure 5.jpeg

Supplementary Table 1.docx

Supplementary Figure 6.jpeg

Supplementary Figure 7.jpeg

Supplementary Figure 1.jpeg

Supplementary Figure 4.jpeg

Supplementary Figure 8.jpeg

Supplementary Figure 9.jpeg

## Data Availability

The data will be made available on reasonable request through the corresponding author.
