# Supplementary material for: Association between anaemia and osteoporosis: a systematic review and meta-analysis
Source: Ann Med. 2026 Jan 6;58(1):2610878. doi: 10.1080/07853890.2025.2610878 (PMC12781946; doi:10.1080/07853890.2025.2610878)
Supplement: Supplemental Material [file IANN_A_2610878_SM3480.docx]

**Caption of supplementary table:**

Supplementary Table 1. Search strategy and keywords used in databases.

**Caption of supplementary figures:**

Supplementary Figure 1. Leave one out sensitivity analysis on the association between anemia and osteoporosis among studies that reported Univariate Odds Ratios.

Supplementary Figure 2. Leave one out sensitivity analysis on the association between anemia and osteoporosis among studies that reported Multivariate Odds Ratios.

Supplementary Figure 3. Subgroup analysis based on geriatric exclusivity among studies that reported Univariate Odds Ratios.

Supplementary Figure 4. Subgroup analysis based on geriatric exclusivity among studies that reported Multivariate Odds Ratios.

Supplementary Figure 5. Subgroup analysis based on anemia type among studies that reported Univariate Odds Ratios.

Supplementary Figure 6. Subgroup analysis based on comorbidities examining the association between anemia and the risk of osteoporosis among studies that reported Multivariate Odds Ratios.

Supplementary Figure 7. Subgroup analysis based on comorbidities examining the association between anemia and the risk of osteoporosis among studies that reported Univariate Odds Ratios.

Supplementary Figure 8. Funnel plot evaluating publication bias on the association between anemia and osteoporosis among studies that reported Univariate Odds Ratios. Egger’s test indicated potential publication bias (p = 0.0179), while Begg’s test was non-significant (p = 0.1124).

Supplementary Figure 9. Subgroup analysis based on the definition of anemia examining the association between anemia and the risk of osteoporosis among studies that reported Univariate Odds Ratios

Supplementary Figure 10. Subgroup analysis based on the definition of anemia examining the association between anemia and the risk of osteoporosis among studies that reported Multivariate Odds Ratios
