# Supplementary figures and images for: Association between anaemia and osteoporosis: a systematic review and meta-analysis
Source: Ann Med. 2026 Jan 6;58(1):2610878. doi: 10.1080/07853890.2025.2610878 (PMC12781946; doi:10.1080/07853890.2025.2610878)

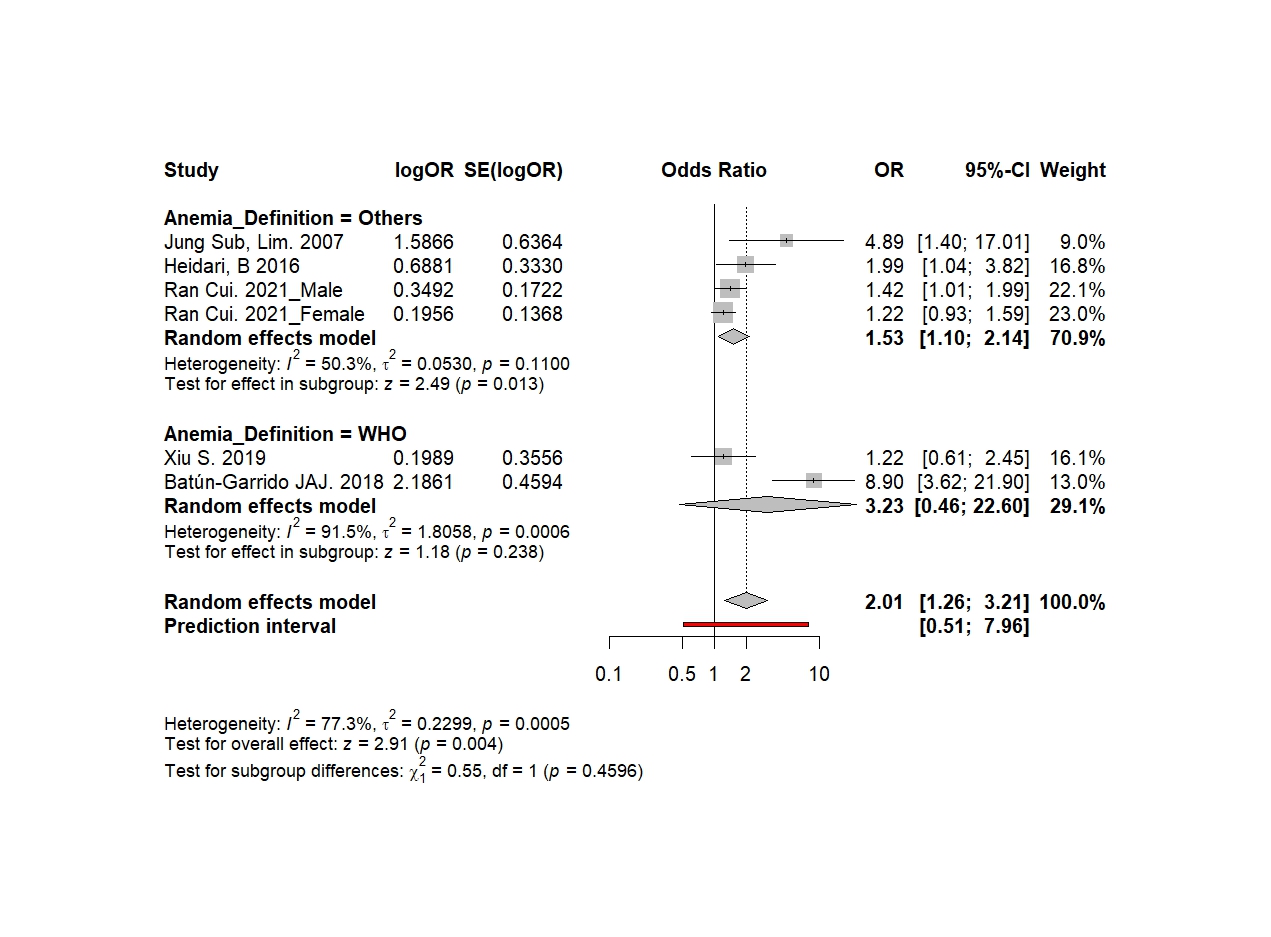

Supplement: Supplementary Figure 10.jpeg [file IANN_A_2610878_SM1859.jpeg]

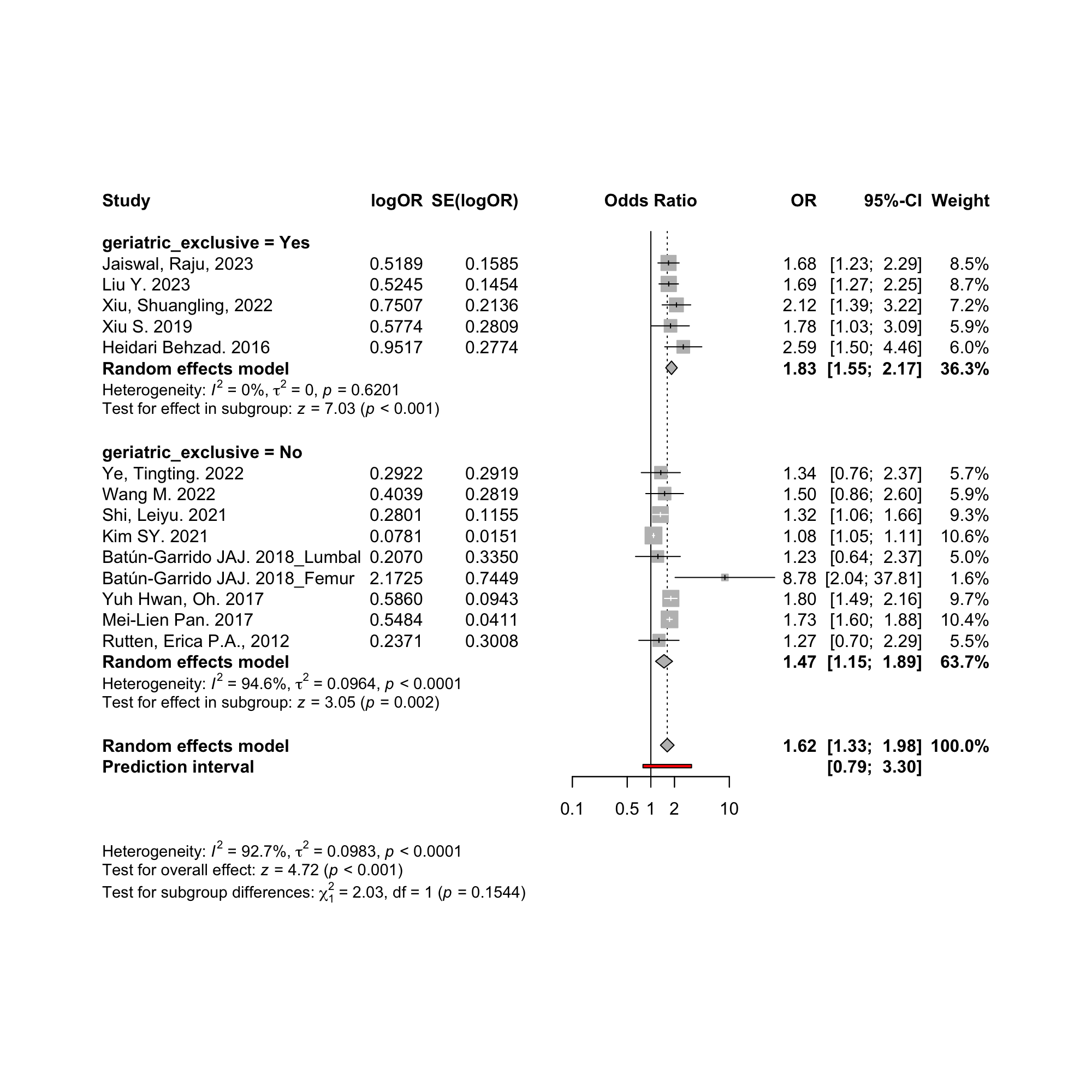

Supplement: Supplementary Figure 3.jpeg [file IANN_A_2610878_SM1858.jpeg]

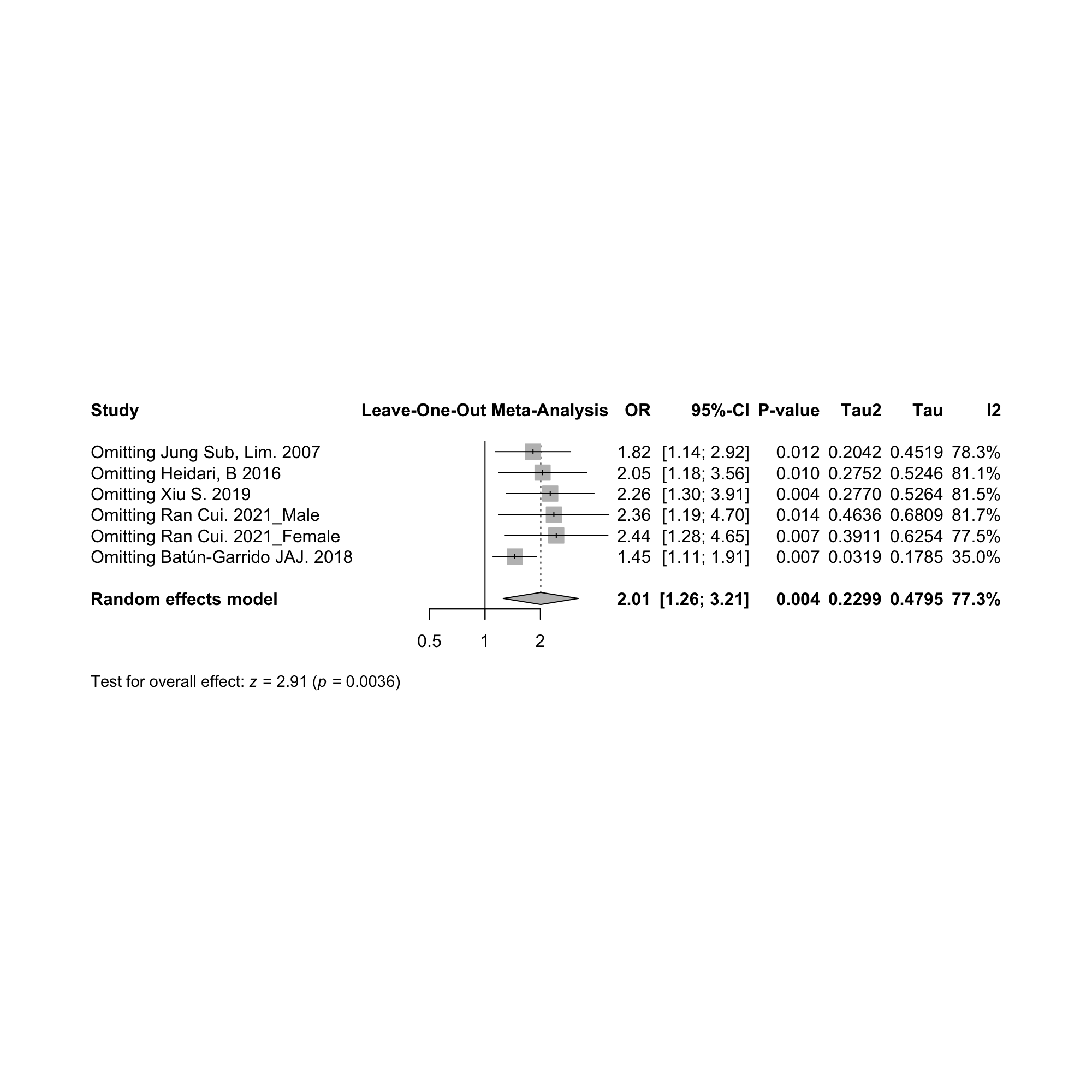

Supplement: Supplementary Figure 2.jpeg [file IANN_A_2610878_SM1857.jpeg]

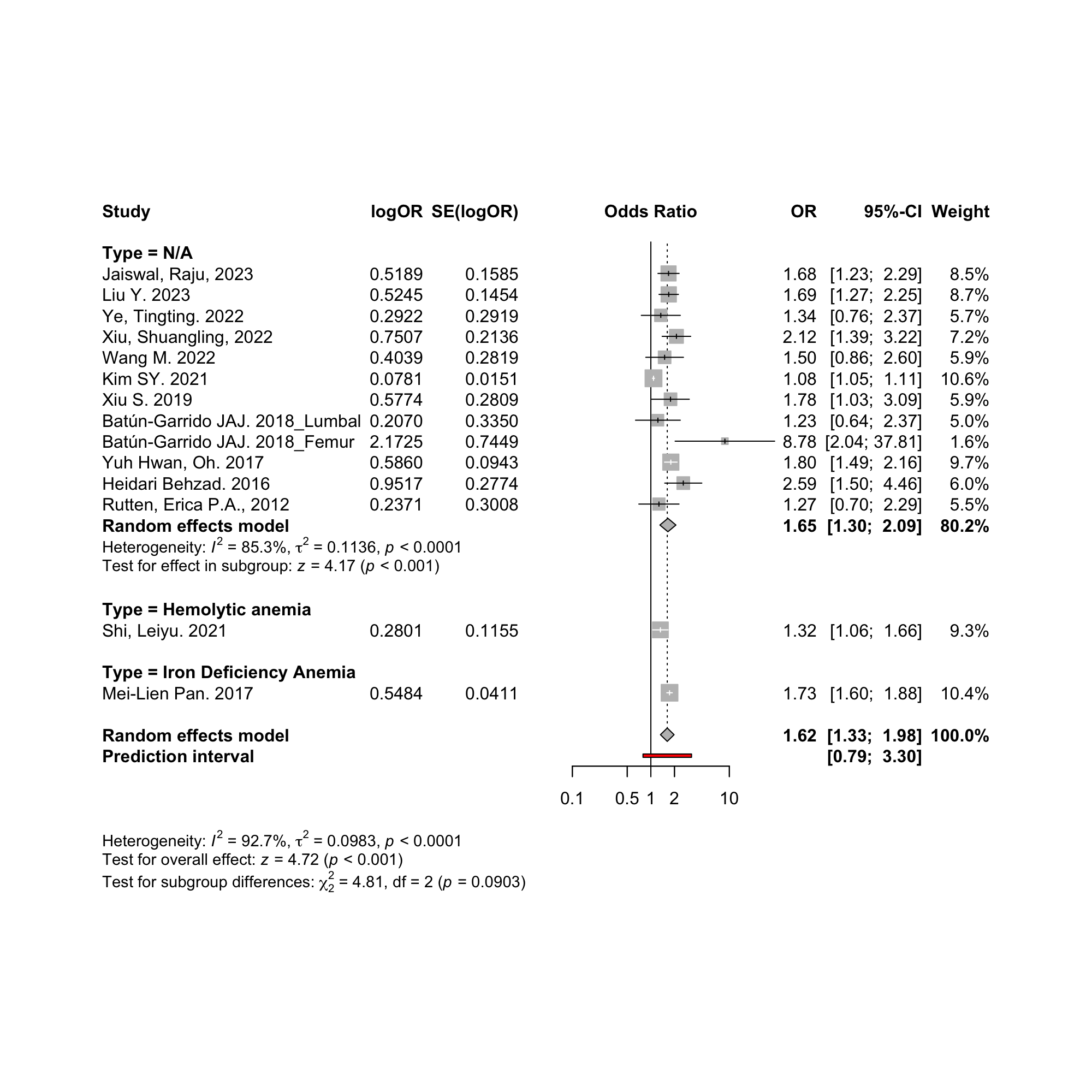

Supplement: Supplementary Figure 5.jpeg [file IANN_A_2610878_SM1855.jpeg]

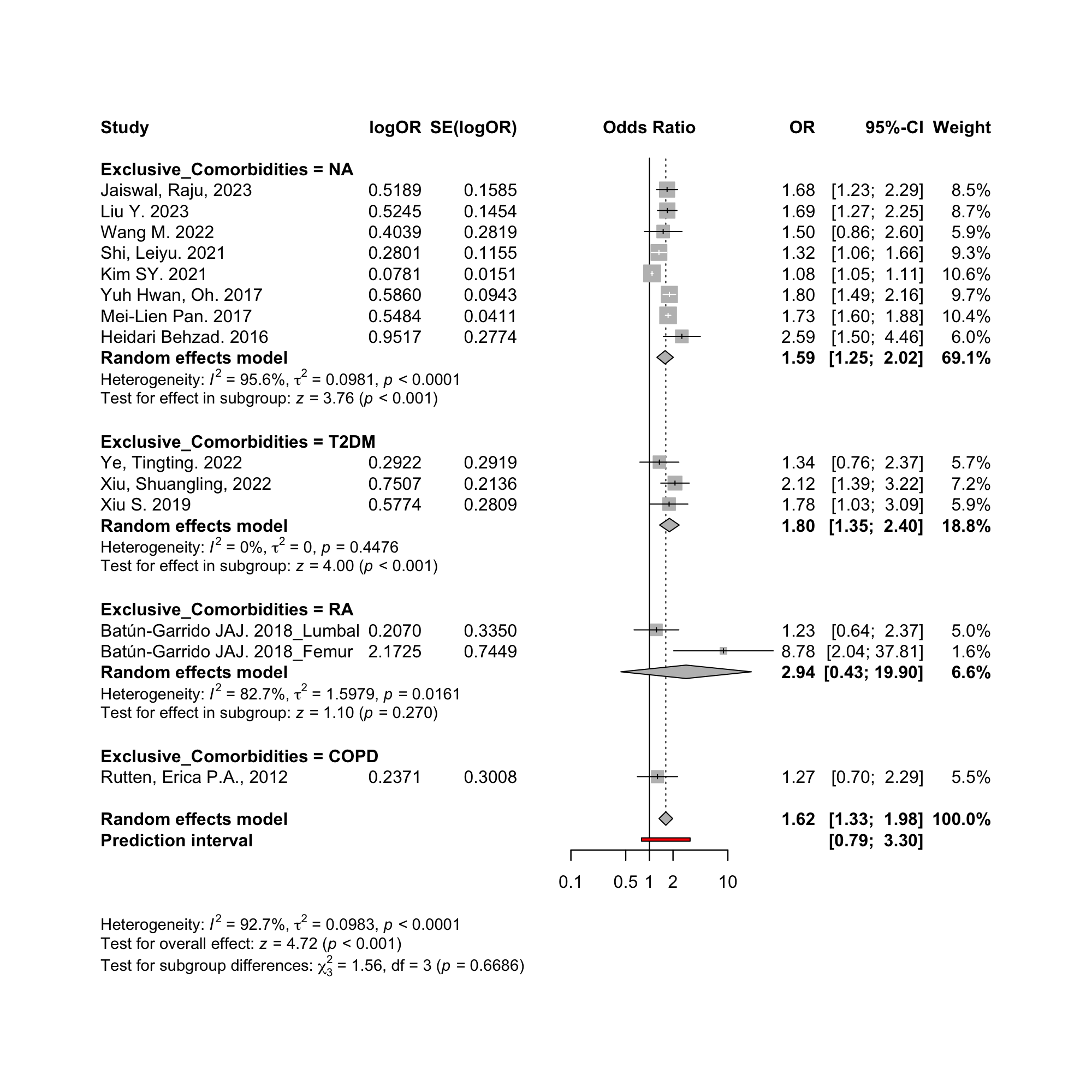

Supplement: Supplementary Figure 6.jpeg [file IANN_A_2610878_SM1853.jpeg]

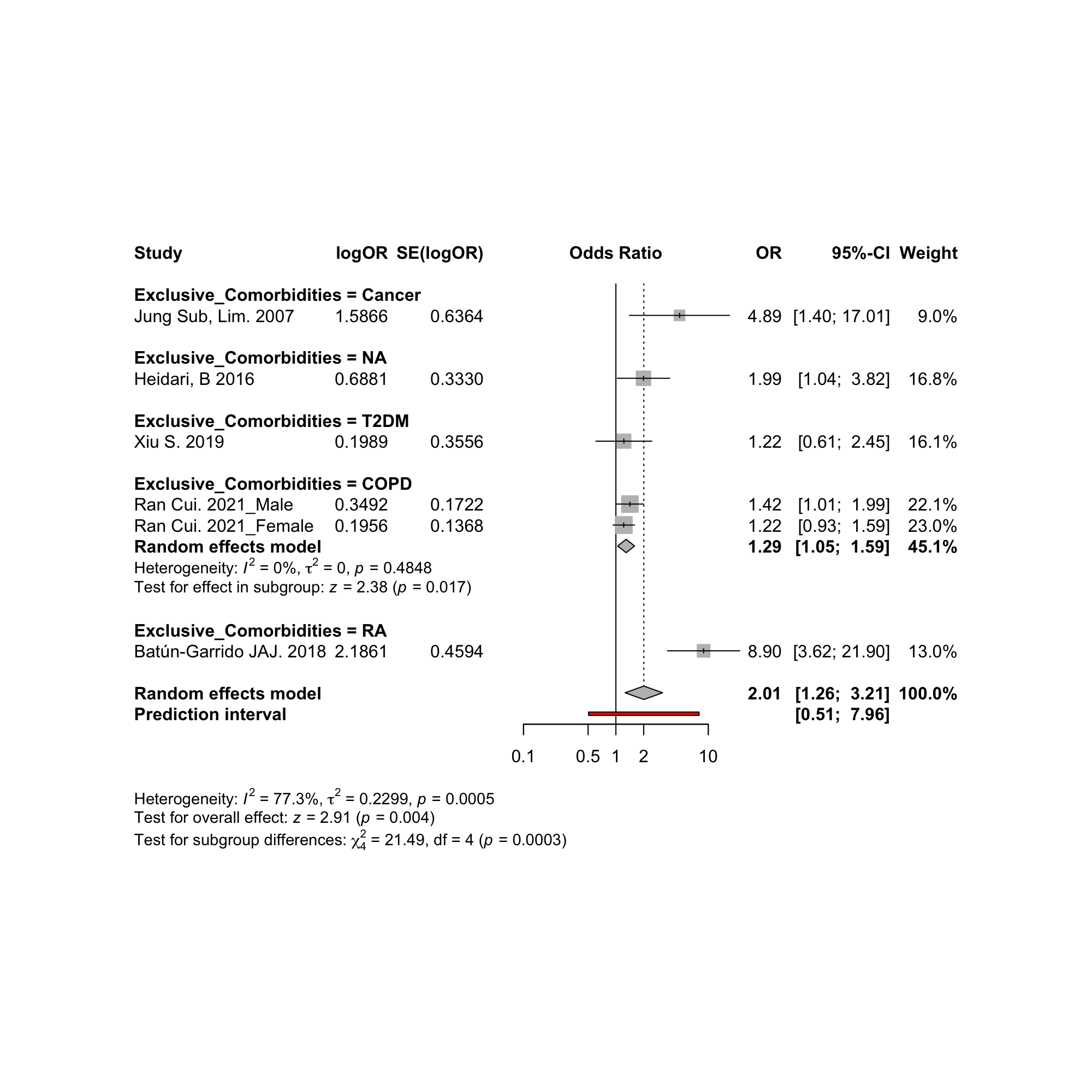

Supplement: Supplementary Figure 7.jpeg [file IANN_A_2610878_SM1852.jpeg]

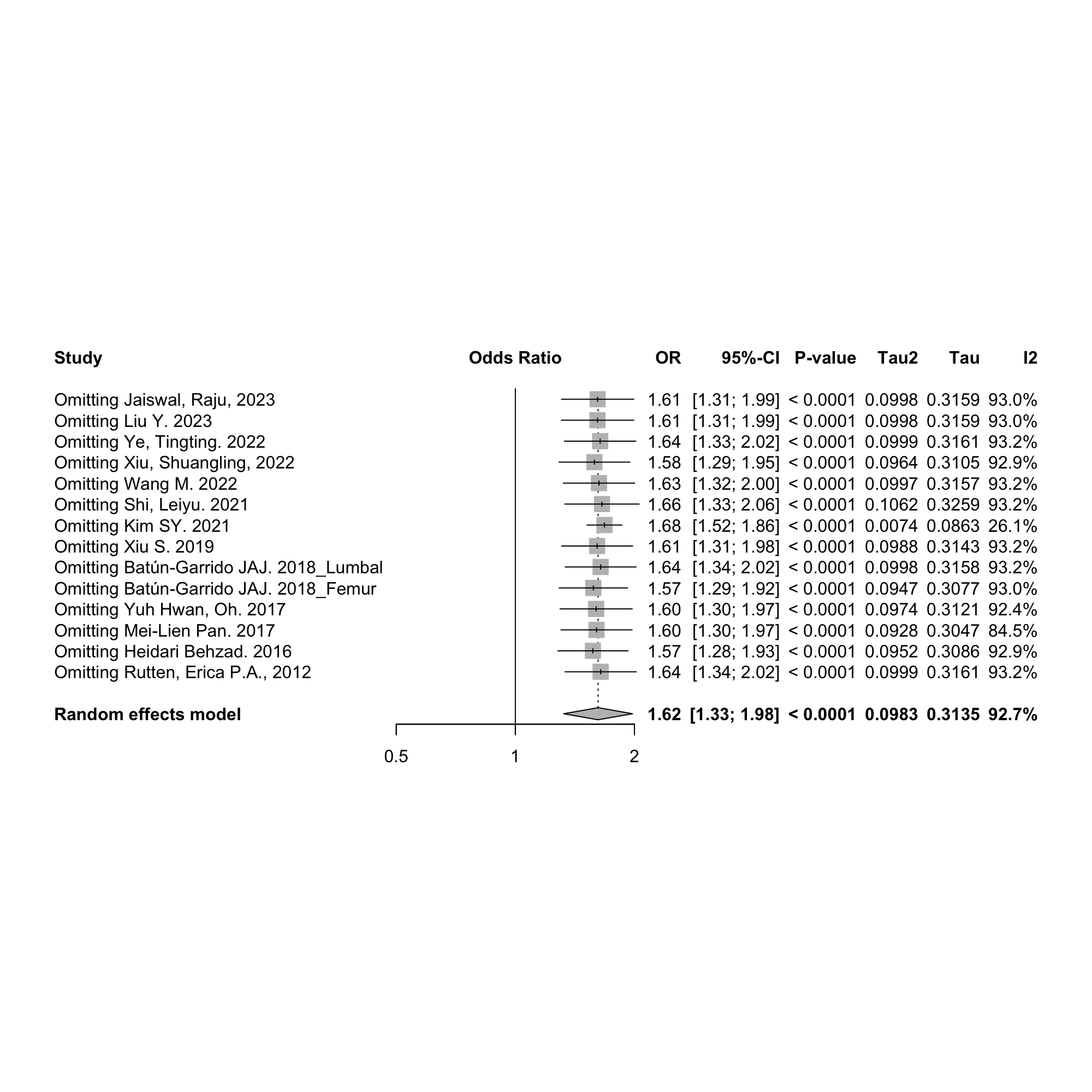

Supplement: Supplementary Figure 1.jpeg [file IANN_A_2610878_SM1851.jpeg]

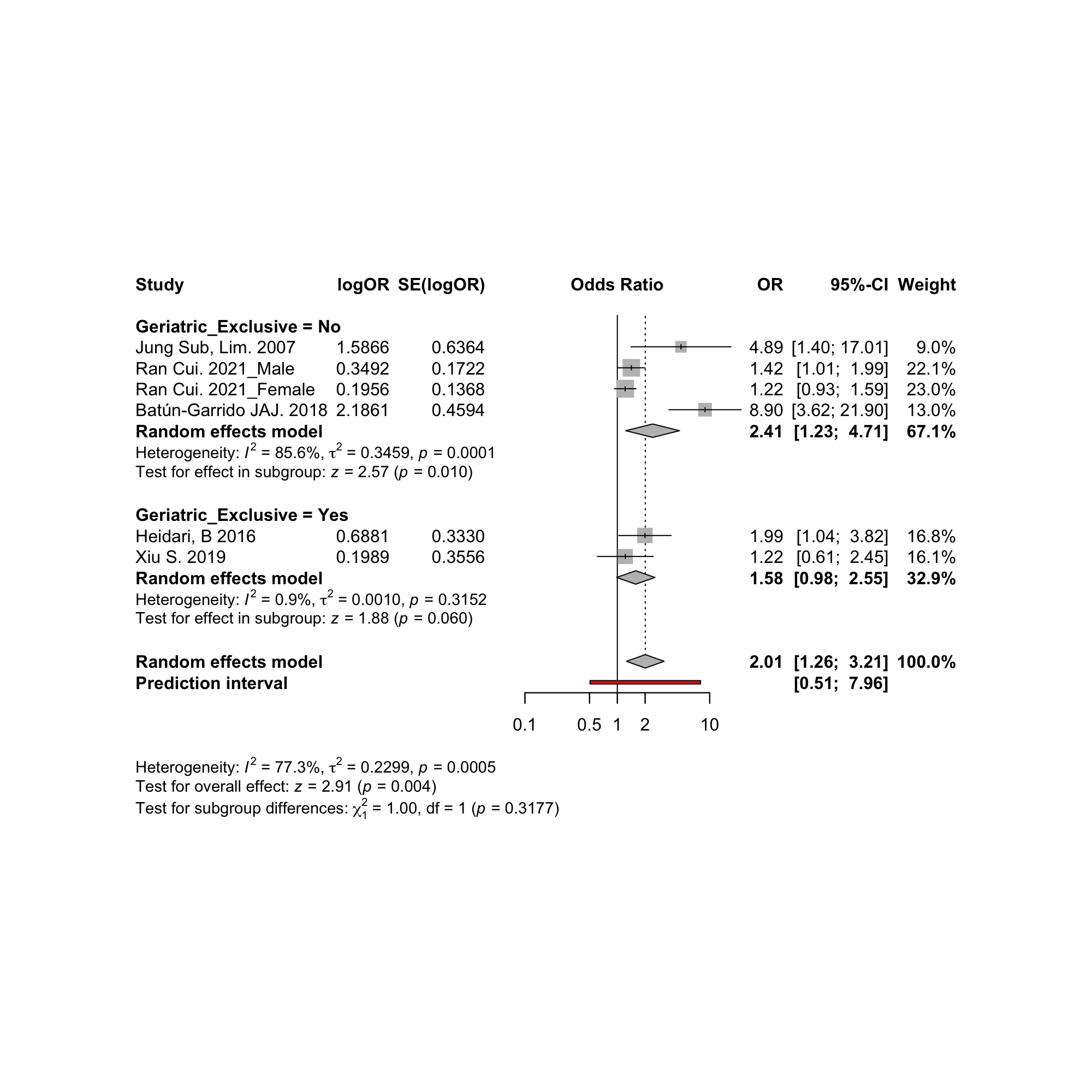

Supplement: Supplementary Figure 4.jpeg [file IANN_A_2610878_SM1850.jpeg]

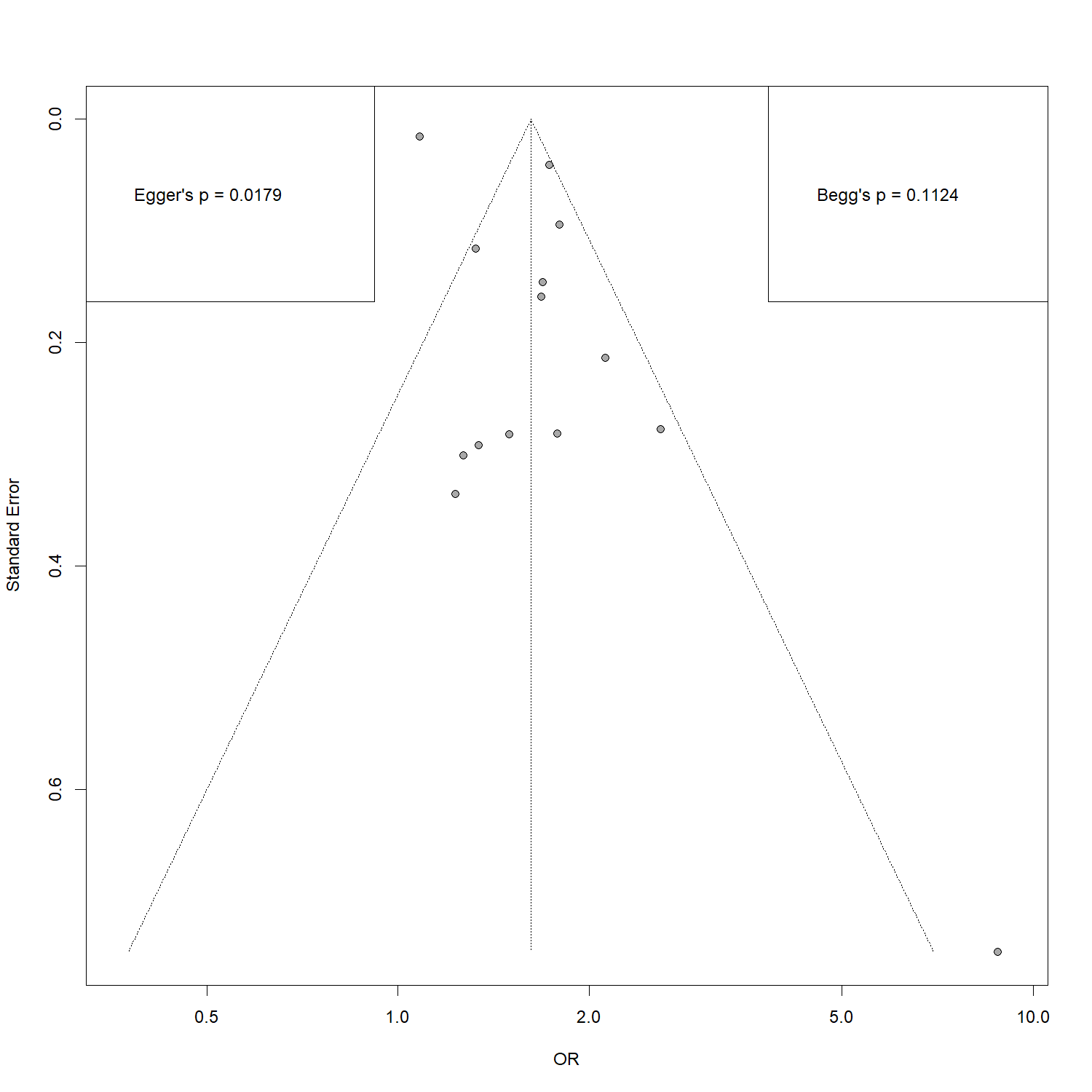

Supplement: Supplementary Figure 8.jpeg [file IANN_A_2610878_SM1849.jpeg]

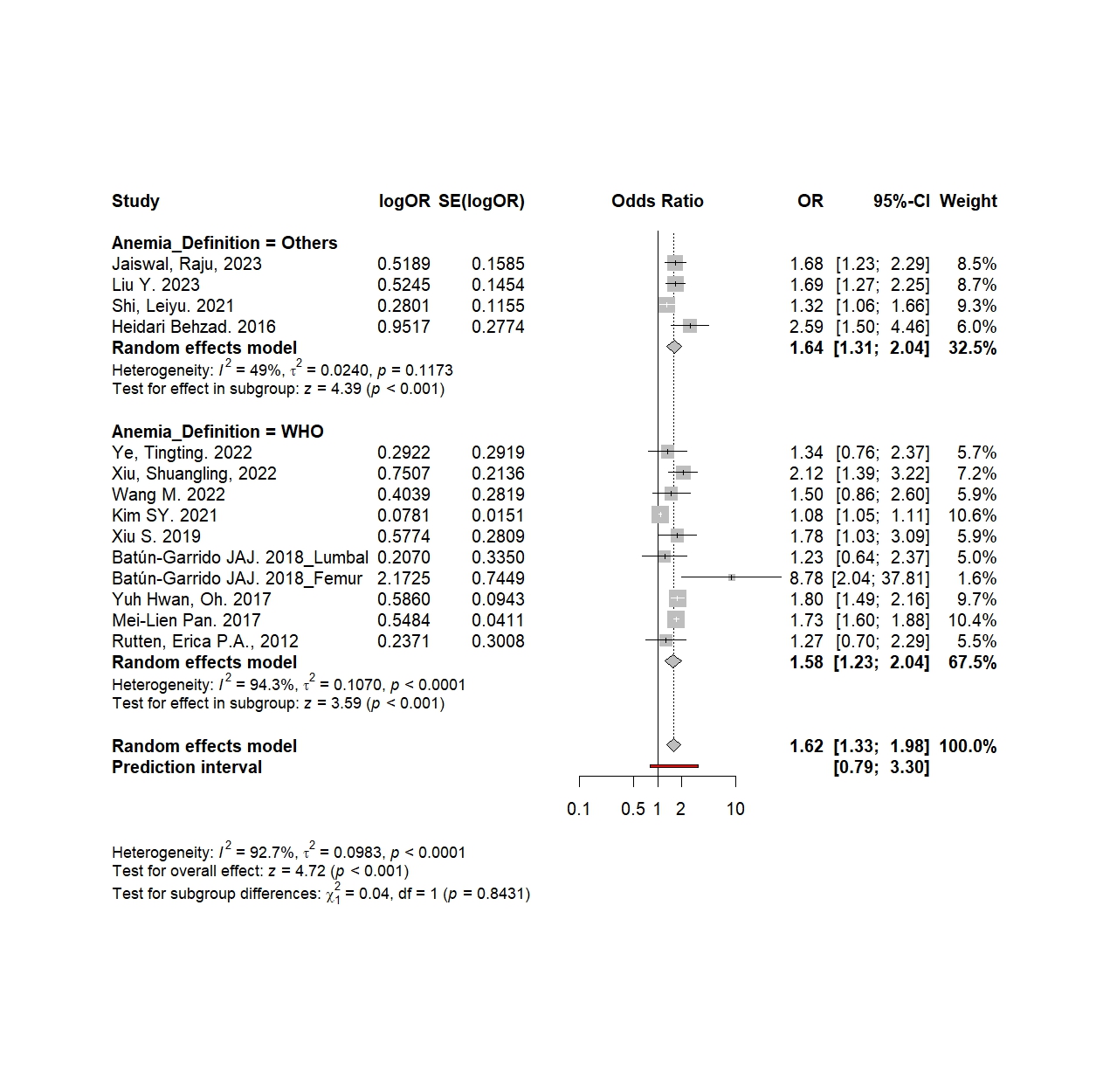

Supplement: Supplementary Figure 9.jpeg [file IANN_A_2610878_SM1848.jpeg]
