# Supplementary material for: Association between anaemia and osteoporosis: a systematic review and meta-analysis
Source: Ann Med. 2026 Jan 6;58(1):2610878. doi: 10.1080/07853890.2025.2610878 (PMC12781946; doi:10.1080/07853890.2025.2610878)
Supplement: Supplementary Table 1.docx [file IANN_A_2610878_SM1854.docx]

**Supplementary Table 1.** Search strategy and keywords used in databases.

| **Database** | **Search Strategy** |
| --- | --- |
| Pubmed | (("osteoporosis"[MeSH Terms]) OR ("osteoporosis"[Title/Abstract]) OR ("osteoporoses"[Title/Abstract]) OR ("age-related osteoporosis"[Title/Abstract]) OR ("age-related osteoporoses"[Title/Abstract]) OR ("age-related bone loss"[Title/Abstract]) OR ("age-related bone losses"[Title/Abstract]) OR ("senile osteoporoses"[Title/Abstract]) OR ("senile osteoporosis"[Title/Abstract]) OR ("post-traumatic osteoporoses"[Title/Abstract]) OR ("post-traumatic osteoporosis"[Title/Abstract])) AND (("anemia"[MeSH Terms]) OR ("anaemia"[Title/Abstract]) OR ("anemia"[Title/Abstract]) OR ("anemias"[Title/Abstract]) OR ("hemoglobins"[MeSH Terms]) OR ("hemoglobins"[Title/Abstract]) OR ("hemoglobin"[Title/Abstract]) OR ("eryhem"[Title/Abstract]) OR ("ferrous hemoglobin"[Title/Abstract]) OR ("haemoglobin" [Title/Abstract])) AND (("risk factors"[MeSH Terms]) OR ("risk factors"[Title/Abstract]) OR ("risk factor"[Title/Abstract]) OR ("population at risk"[Title/Abstract]) OR ("populations at risk"[Title/Abstract]) OR ("risk scores"[Title/Abstract]) OR ("risk score"[Title/Abstract]) OR ("risk factor scores"[Title/Abstract]) OR ("health correlates"[Title/Abstract]) OR ("social risk factor"[Title/Abstract]) OR ("risk"[Title/Abstract]) OR ("predictors"[Title/Abstract]) OR ("predictor"[Title/Abstract])) |
| Scopus | TITLE-ABS-KEY ( {osteoporosis} OR {osteoporoses} OR {age-related osteoporosis} OR {age-related osteoporoses} OR {age-related bone loss} OR {age-related bone losses} OR {senile osteoporosis} OR {senile osteoporoses} OR {post-traumatic osteoporosis} OR {post-traumatic osteoporoses} ) AND TITLE-ABS-KEY ( {anemia} OR {anaemia} OR {anemias} OR {hemoglobins} OR {hemoglobin} OR {eryhem} OR {ferrous hemoglobin} OR {haemoglobin} ) AND TITLE-ABS-KEY ( {risk factors} OR {risk factor} OR {population at risk} OR {populations at risk} OR {risk scores} OR {risk score} OR {risk factor scores} OR {health correlates} OR {social risk factor} OR {risk} OR {predictors} OR {predictor} ) |
| Ebsco | AB ((("osteoporosis" OR "osteoporoses" OR "age-related osteoporosis" OR "age-related osteoporoses" OR "age-related bone loss" OR "age-related bone losses" OR "senile osteoporoses" OR "senile osteoporosis" OR "post-traumatic osteoporoses" OR "post-traumatic osteoporosis"))) AND AB ((("anaemia" OR "anemia" OR "anemias" OR "hemoglobins" OR "hemoglobin" OR "eryhem" OR "ferrous hemoglobin" OR "haemoglobin"))) AND AB ((("risk factors" OR "risk factor" OR "population at risk" OR "populations at risk" OR "risk scores" OR "risk score" OR "risk factor scores" OR "health correlates" OR "social risk factor" OR "risk" OR "predictors" OR "predictor")))  AB ((("osteoporosis" OR "osteoporoses" OR "age-related osteoporosis" OR "age-related osteoporoses" OR "age-related bone loss" OR "age-related bone losses" OR "senile osteoporoses" OR "senile osteoporosis" OR "post-traumatic osteoporoses" OR "post-traumatic osteoporosis"))) AND AB ((("anaemia" OR "anemia" OR "anemias" OR "hemoglobins" OR "hemoglobin" OR "eryhem" OR "ferrous hemoglobin" OR "haemoglobin"))) AND AB ((("risk factors" OR "risk factor" OR "population at risk" OR "populations at risk" OR "risk scores" OR "risk score" OR "risk factor scores" OR "health correlates" OR "social risk factor" OR "risk" OR "predictors" OR "predictor"))) |
| Science Direct | ({osteoporosis} OR {osteoporoses}) AND ({anemia} OR {anaemia} OR {hemoglobin} OR {haemoglobin) AND ({risk factor} OR {risk factors} OR {population at risk}) |
